# Activation of KEAP1/NRF2/P62 signaling alleviates high phosphate-induced calcification of vascular smooth muscle cells by suppressing reactive oxygen species production

**DOI:** 10.1038/s41598-019-46824-2

**Published:** 2019-07-17

**Authors:** Ran Wei, Mayu Enaka, Yasuteru Muragaki

**Affiliations:** Department of Pathology, Wakayama Medical University School of Medicine, Wakayama, Japan

**Keywords:** Cardiovascular models, Stress signalling

## Abstract

Vascular calcification is a complication of diseases and conditions such as chronic kidney disease, diabetes, and aging. Previous studies have demonstrated that high concentrations of inorganic phosphate (Pi) can induce oxidative stress and vascular smooth muscle cell calcification. KEAP1 (Kelch-like ECH-associated protein 1)/NF-E2-related factor 2 (NRF2) signaling has been shown to play important roles in protecting cells from oxidative stress. The current study aims to investigate the possible involvement of the KEAP1/NRF2/P62 -mediated antioxidant pathway in vascular calcification induced by high Pi levels. Exposure of vascular smooth muscle cells (VSMCs) to high Pi concentrations promoted the accumulation of reactive oxygen species (ROS) and the nuclear translocation of NRF2, along with an increase in P62 levels and a decrease in KEAP1 levels. A classic NRF2 activator, tert-butylhydroquinone (tBHQ), significantly decreased ROS levels and calcium deposition in VSMCs by promoting the nuclear translocation of NRF2 and upregulating P62 and KEAP1 expression. In contrast, silencing NRF2 and P62 with siRNAs increased the levels of ROS and calcium deposition in VSMCs. In conclusion, VSMC calcification can be alleviated by the activation of the KEAP1/NRF2/P62 antioxidative pathway, which could have a protective role when it is exogenously activated by tBHQ.

## Introduction

Arterial calcification is a common complication of chronic kidney disease (CKD), atherosclerosis, diabetes, and chronic heart failure^1–4^. It usually occurs in the tunica intima and tunica media of the artery. Calcification in the arterial media not only leads to vascular stiffening but also is a major risk factor for cardiovascular mortality and morbidity^5,6^. It has already been found that vascular calcification is an active cell-mediated process that occurs in response to abnormal environmental cues, including oxidative stress, inflammatory cytokines, alterations in the extracellular matrix and increased calcium and phosphate levels, similar to bone development^3,7–9^. During this process, the differentiated vascular smooth muscle cells (VSMCs) undergo dedifferentiation and subsequently undergo osteogenic transition, which results in vascular calcification^10^. Therefore, explorations of the mechanisms underlying the osteogenic and chondrogenic reprogramming of VSMCs will provide novel targets and new strategies for the clinical prevention of vascular calcification.

Oxidative stress is reported to be an important mediator of the osteochondrogenic transdifferentiation of VSMCs and is closely associated with the development of cardiovascular disease and vascular calcification *in vitro* and *in vivo* in patients with CKD^7,11–14^. In the presence of oxidative stress, reactive oxygen species (ROS) cause lipid peroxidation, serious damage to DNA and proteins, and other abnormal biochemical changes^15^. Mammalian cells have evolved cytoprotective mechanisms that counteract ROS generation via the regulation of Kelch-like ECH-associated protein 1/NF-E2-related factor 2 (KEAP1/NRF2) signaling^16,17^. In the canonical KEAP1/NRF2 pathway, NRF2 is suppressed by the negative regulator KEAP1 under normal conditions, which leads to its ubiquitylation and proteasomal degradation. As a result of changes in the intracellular redox balance, KEAP1 is inactivated via the modification of cysteine residues and loses its ability to interact with NRF2. As a consequence, NRF2 dissociates from the KEAP1-NRF2 complex and translocates into the nucleus, where it regulates the expression of antioxidant and anti-inflammatory genes via consensus cis-elements known as antioxidant response elements (ARE)^18^. Several noncanonical pathways resulting in KEAP1/NRF2 activation involve the competitive inhibition of the KEAP1-NRF2 interaction by intracellular proteins such as P62/Sqstm1^19^, PGAM5^20^, and CIP/WFAI^21^, among which the best characterized pathway is the KEAP1/NRF2/P62 axis.

P62/SQSTM1 is a multifunctional stress-inducible scaffold protein and a marker of autophagic degradation that is involved in cell signaling, oxidative stress, and autophagy^22^. According to recent studies, P62 regulates the KEAP1/NRF2 pathway^19,23–26^. Under physiological conditions, P62 has been shown to sequester KEAP1 into autophagosomes, which impairs the ubiquitylation of NRF2 and leads to the release of NRF2 into the nucleus to induce the transcription of numerous cytoprotective genes^27^. The P62 promoter contains an ARE that is bound by NRF2 and that activates the transcription of the P62 gene, which implies that a positive feedback loop exists within the KEAP1/NRF2//P62 axis^25^. Therefore, P62 contributes to the capacity of cells to defend themselves against oxidative stress.

To the best of our knowledge, the interaction between vascular calcification induced by high Pi levels and the KEAP1/NRF2/P62 pathway remains unclear. In the present study, we aimed to determine the possible involvement of the KEAP1/NRF2/P62 antioxidant pathway in VMSC calcification induced by high Pi levels.

## Results

### High Pi concentrations promote oxidative stress and calcification of VSMCs

**Figure 1:**
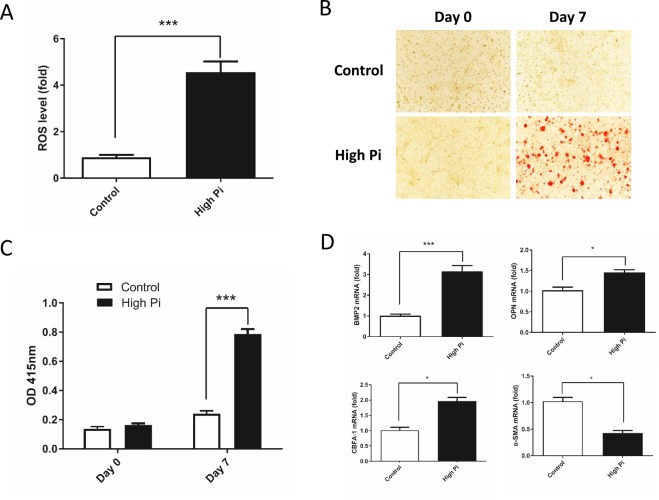
High phosphate concentrations promoted oxidative stress and calcification of VSMCs. (**A**) VSMCs were exposed to high-Pi medium or control medium for 48 h, and the ROS levels were determined with DCFH-DA, which was used to measure the levels of ROS within cells. (P = 0.0004) (**B**) VSMCs were cultured in either control or high-Pi medium for 7 days. Alizarin red S staining was performed to visualize calcification. (**C**) Alizarin red S was dissolved in 0.5 N HCl and 5% SDS, and the absorbance was measured at 415 nm to quantify the calcium content. Note that there was a significant increase in cultured VSMCs in high-Pi medium at day7 compared with those in the control group. (P = 0.000028) (**D**) The expression of BMP2, CBFA-1, osteopontin and α-SMA mRNAs were assessed using real-time PCR. The bone marker genes BMP2, CBFA-1, osteopontin were upregulated (P = 0.000076, 0.0365, and 0.0206, respectively), while the VSMC marker α-SMA was downregulated (P = 0.0154). The data were normalized to GAPDH (n = 4, *P < 0.05, ***P < 0.001, mean ± SD).

First, we used DCFH-DA, which is a fluorogenic dye that measures ROS activity within cells, to investigate whether high Pi concentrations directly increase the production of ROS in VSMCs. VSMCs exposed to high Pi medium exhibited a 4.3-fold increase in fluorescence intensity compared with that of control cells (Figure 1A). VSMC calcification, as indicated by Alizarin red S staining, was dramatically increased in the cells exposed to high Pi concentrations for 7 days, while mineral deposition was not detected in cells cultured in control medium (Figure 1B,C). These results were further confirmed by von Kossa staining (Suppl. Figure 1). In addition, we investigated the effect of high Pi concentrations on the osteogenic differentiation of VSMCs by examining the expression of bone marker genes such as BMP2, OPN, and CBFA-1. High Pi concentrations increased the levels of BMP2 mRNA, OPN mRNA, and CBFA-1 mRNA (Figure 1D). On the other hand, the level of the α-SMA mRNA, which is a specific VSMC marker, was significantly decreased by high Pi concentrations compared with that found in the control group (Figure 1D).

### High Pi concentrations induce the transcriptional activation of NRF2 and promote the nuclear translocation of NRF2

**Figure 2:**
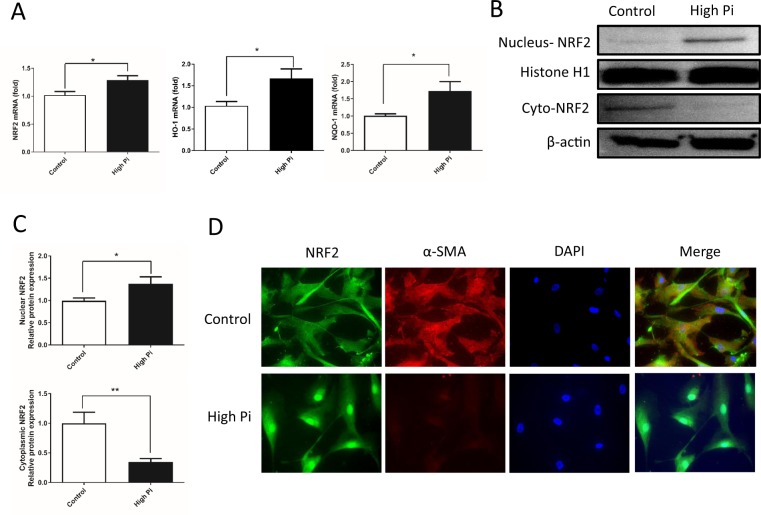
High Pi concentrations induced the transcriptional activity of NRF2 and promoted its nuclear translocation. VSMCs were cultured in high-Pi medium for 7 days. (**A**) The expression of NRF2 and its downstream target genes NQO-1 and HO-1 was examined using real-time PCR. Expression was normalized to GAPDH. High Pi concentrations increased the mRNA levels of NRF2 (P = 0.0161), NQO-1 (P = 0.0201) and HO-1 (P = 0.0192). (**B**) Western blot showing the translocation of NRF2 from the cytoplasm to the nucleus with a specific antibody. β-actin and histone 1 were used as loading controls for the cytoplasmic or nuclear fractions, respectively. (**C**) Densitometric quantification of the NRF2 protein in the blots in (**B**) is shown. (P = 0.0493, and 0.0075, respectively) (**D**) Immunofluorescence staining was also used to show the nuclear translocation of NRF2 when VSMCs were exposed to high Pi concentrations. VSMCs were stained with specific primary antibodies against NRF2 and α-SMA. (n = 3, *P < 0.05, **P < 0.01, mean ± SD).

We observed the effects of high Pi concentrations on the activation of NRF2, which is the main critical transcription factor that regulates antioxidant responses, to identify the critical antioxidant signals involved in vascular calcification. Based on quantitative PCR data, high Pi concentrations increased the levels of the NRF2 transcript and those of its specific target genes NQO-1 and HO-1 (Figure 2A). Western blot analyses and densitometric quantification of the bands showed that high Pi concentration decreased the cytoplasmic level of the NRF2 protein and increased the nuclear level of the NRF2 protein (Figure 2B, C). The results were further confirmed using immunofluorescence staining, which showed the nuclear translocation of NRF2 and the decreased levels of α-SMA in VSMCs that had been incubated with high concentrations of Pi for 7 days (Figure 2D). Based on these data, high Pi concentrations stimulate antioxidant activity by increasing NRF2 expression and promoting its nuclear translocation.

### High Pi concentrations affect the expression of KEAP1 and P62 during VSMC calcification

**Figure 3:**
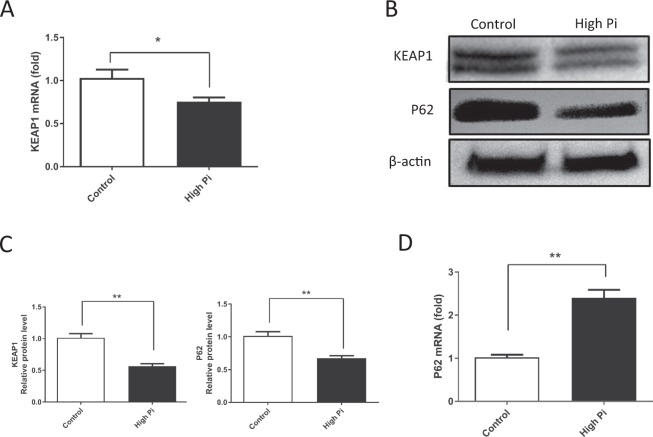
Expression of KEAP1 and P62 during VSMC calcification induced by high Pi concentrations. (**A**,**D**) The expression of the KEAP1 (P = 0.0215) and the P62 mRNAs (P = 0.0049) was determined using real-time PCR (n = 4). (**B**) Protein extracts were prepared and analyzed by Western blot using anti-KEAP1, P62, and β-actin antibodies. (**C**) Quantitative data showing the KEAP1 (P = 0.0016) and P62 (P = 0.0022) levels. (n = 3, *P < 0.05, **P < 0.01, mean ± SD).

Under normal conditions, NRF2 is degraded by ubiquitination that is mediated by KEAP1^28^. P62, which is also known as sequestosome 1 (Sqstm1), competes with NRF2 for binding to KEAP1^29^. We therefore analyzed the expression of KEAP1 and P62 during VSMC calcification. High Pi concentrations decreased KEAP1 expression in VSMCs at both the mRNA and protein levels (Figure 3A–C and Suppl. Figure 2A). Interestingly, in the presence of high Pi concentrations, the expression of the P62 and NRF2 mRNAs was upregulated, while the levels of the P62 protein were decreased in VSMCs, as shown via Western blottings and immunofluorescence staining (Figure 3B–D and Suppl. Figure 2B). These results indicate that high Pi concentrations may increase NRF2 expression, upregulate P62 at the transcriptional level, and downregulate KEAP1.

### tBHQ attenuates oxidative stress and calcification in VSMCs

**Figure 4:**
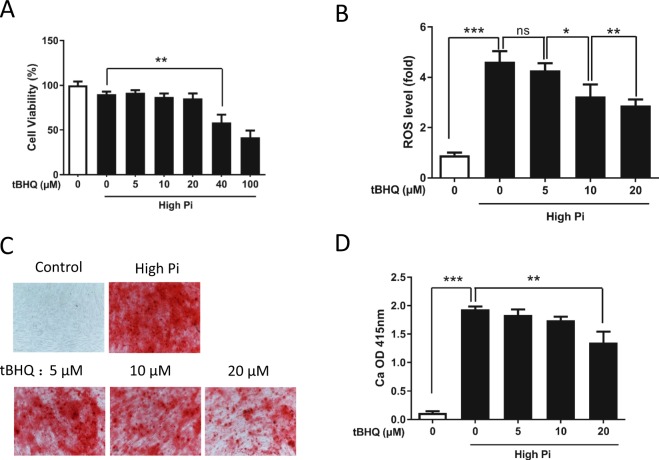
The classic NRF2 activator tBHQ attenuated high Pi concentration-induced oxidative stress and VSMC calcification. VSMCs were cultured in control medium or high-Pi medium supplemented with progressively increasing concentrations of tBHQ (5–100 μM) to measure its effects on the prevention of oxidative stress and VSMC calcification. (**A**) VSMCs were incubated with control medium or high-Pi medium supplemented with tBHQ (5–100 μM) for 48 h. After treatment, the number of surviving cells was determined using a CCK-8 assay. At a concentration of 40 μM, there was a significant decrease in cell viability. (P = 0.0056). (**B**) Intracellular ROS levels were measured using DCFH-DA staining and a microplate reader after VSMCs were cultured for 48 h. The P value of the comparison of cells exposed to high Pi concentrations with control celsls was 0.000023. The P values for cells exposed to different concentrations of tBHQ (5, 10, and 20 μM) compared with cells cultured only in high Pi concentrations ns, 0.0328, and 0.0078, respectively. (**C**) Alizarin red S staining was performed to visualize VSMC calcification at day 7 after treatment with or without tBHQ (5–100 μM). (**D**) Quantification of calcium deposition showed that there was a significant decrease in calcification at a concentration of tBHQ (20 μM) (P = 0.0046), while there was a dramatic increase in calcification in the high-Pi group (P = 0.000019) compared with the control group. (n = 3, *P < 0.05, **P < 0.01, ***P < 0.001, mean ± SD).

We used a classic activator of NRF2, tBHQ, which is commonly used to stimulate the NRF2 pathway^30^, to further elucidate the role of the KEAP1/NRF2/P62 pathway in VSMC calcification induced by high concentrations of Pi. First, we investigated the effect of tBHQ on cytotoxicity. VSMCs were incubated in calcification media in the presence of tBHQ (5–100 μM). We found that a concentration of tBHQ between 40–100 μM increased cell toxicity (Figure 4A). Quantification of ROS production by measuring DCFH-DA fluorescence intensity showed that tBHQ significantly reduced intracellular ROS production in VSMCs cultured in the presence of high Pi concentrations (Figure 4B). Next, we examined the degree of VSMC calcification induced by oxidative stress in response to high Pi concentrations. The level of calcium deposition in VSMCs exposed to high Pi concentrations was dramatically increased, while this increase in calcification was ameliorated by tBHQ treatment; the most effective concentration of tBHQ was 20 μM (Figure 4C, D). Twenty micromolar tBHQ was considered to be a proper concentration that produced maximum effects and was used in subsequent experiments. These data suggest that tBHQ can suppress VSMC calcification and ameliorate oxidative stress in VSMCs.

### tBHQ ameliorates VSMC calcification by inducing NRF2 nuclear translocation and increasing P62 and KEAP1 expression

**Figure 5:**
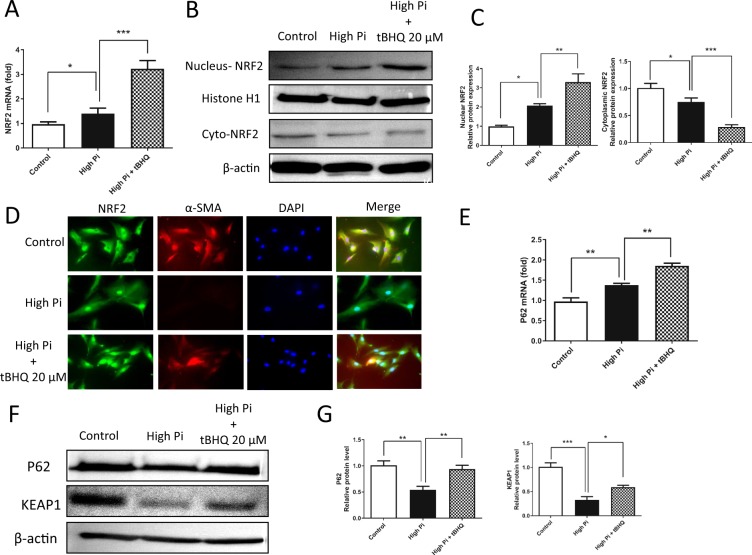
tBHQ ameliorates VSMC calcification by inducing the nuclear translocation of NRF2 and increasing P62 and KEAP1 expression. VSMCs were cultured in control or high-Pi medium with or without tBHQ (20 μM) for 7 days. (**A**,**E**) The expression of the NRF2 (P = 0.0002 and 0.0440, respectively) and P62 mRNAs (P = 0.0068 and 0.0024, respectively) was examined by real-time PCR and shown to be significantly upregulated by tBHQ (20 μM) when compared with the high-Pi or control groups. (**B**) The translocation of NRF2 from the cytoplasm to the nucleus was detected by Western blotting using specific antibodies. β-actin and histone 1 were used as loading controls for the cytoplasmic and nuclear fractions, respectively. (**C**) Densitometric quantification of nuclear (P = 0.0114 and 0.0257, respectively) and cytoplasmic (P = 0.0007 and 0.0469, respectively) NRF2 protein in the Western blots in B is shown. (**D**) NRF2 localization was observed by immunofluorescence staining using specific primary antibodies against NRF2 and α-SMA. (**F**) Levels of the P62 and KEAP1 proteins were detected by Western blot using anti-P62 and anti-KEAP1 antibodies. β-actin was used as a loading control. (**G**) Densitometric quantification of the P62 (P = 0.0014, 0.0052) and KEAP1 proteins (P = 0.0332, 0.000029) in the blots in (**F**) is shown.

We observed whether tBHQ inhibited VSMC calcification by activating the KEAP1/NRF2/P62 signaling pathway. The expression and nuclear translocation of NRF2 were significantly increased in response to tBHQ (20 μM) in the presence of high Pi concentrations compared with those of the high-Pi and control groups (Figure 5A–D). In addition, the expression of the target genes of NRF2, such as HO-1 and NQO-1, was also increased (Suppl. Figure 3A). Moreover, the expression of P62 and the level of KEAP1 protein were significantly increased following tBHQ treatment (20 μM) (Figure 5E–G). However, the KEAP1 mRNA level was not significantly changed (Suppl. Figure 3B).

### Knockdown of NRF2 and P62 aggravates oxidative stress and VSMC calcification induced by high Pi concentrations

**Figure 6:**
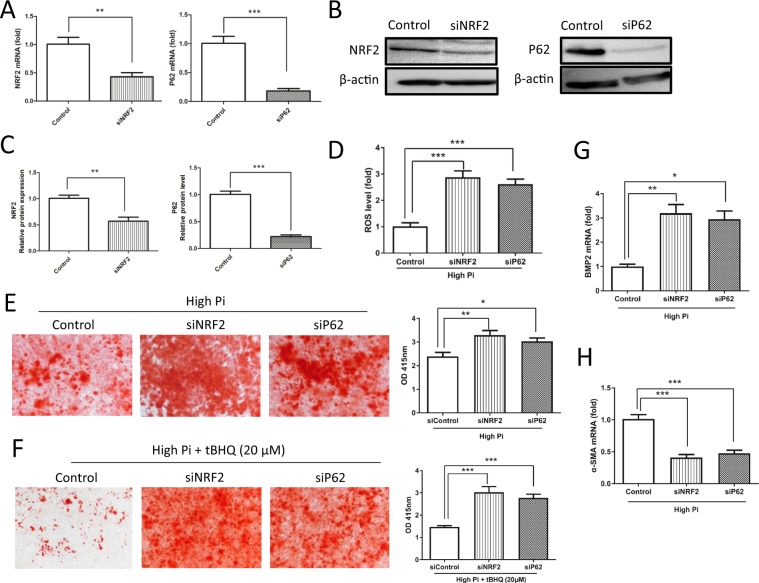
Knockdown of NRF2 and P62 exacerbates oxidative stress and VSMC calcification induced by high Pi concentrations. VSMCs were transiently transfected with control, NRF2 or P62 siRNAs for 72 h and cultured in high-Pi medium with or without tBHQ (20 μM). (**A**,**B**) The levels of the NRF2 and P62 mRNAs (P = 0.0020 and 0.000018, respectively) and proteins were analyzed using real-time PCR and Western blotting, respectively. (**C**) Densitometric quantification of NRF2 and P62 proteins shown in (**B**) (P = 0.0010 and 0.000005, respectively). (**D**) Comparison of ROS levels between cells transfected or not transfected with NRF2 or P62 siRNA for 72 h. The ROS levels were analyzed using DCFH-DA staining (P = 0.00021, 0.00046, respectively). After gene silencing, VSMCs were cultured in high-Pi medium with or without tBHQ (20 μM) for 7days. Mineral deposition in VSMCs and quantification of the calcium content were determined using Alizarin red S staining. (**E**) The levels of calcium deposition in NRF2- (P = 0.0052) or P62-silenced (P = 0.0439) groups was significantly increased compared with that in siControl group. (**F**) tBHQ lost its inhibitory effects on calcification in the NRF2- (P = 0.00014) or P62-silenced (P = 0.0003) groups compared with the siControl group. (**G** and **H**) The expression of the BMP2 (P = 0.0037 and 0.0170, respectively) and α-SMA mRNAs (P = 0.000062 and 0.00023, respectively) was determined using real-time PCR (n = 3, *P < 0.05, **P < 0.01, and ***P < 0.001, mean ± SD).

Next, we performed siRNA-mediated silencing of the NRF2 and P62 genes in VSMCs to clarify the roles of the KEAP1/NRF2/P62 pathway in oxidative stress and VSMC calcification. The levels of NRF2 and P62 mRNAs and proteins were significantly decreased in cells transfected with NRF2 and P62 siRNAs compared with control cells (Figure 6A–C). The low levels of ROS were obviously increased in VSMCs transfected with siRNAs against both NRF2 and P62 compared with the control groups (Figure 6D). In addition, the amount of cell mineralization was increased in the transfected cells compared with the control cells. In the transfected cells, the anticalcification effect of tBHQ was abolished (Figure 6E, F). Additionally, real-time PCR analysis indicated a significant increase in the expression of the specific osteogenic marker BMP2 and a significant decrease in the expression of the VSMC marker α-SMA in cells transfected with NRF2 or P62 siRNAs (Figure 6G, H). These results indicate that tBHQ exerts a protective effect against oxidative stress and VSMC calcification via further activation of the KEAP1/NRF2/P62 pathway and that knockdown of NRF2 or P62 exacerbates calcification induced by high Pi levels.

### The role of KEAP1/NRF2/P62 in VSMC calcification induced by high Pi concentrations

**Figure 7:**
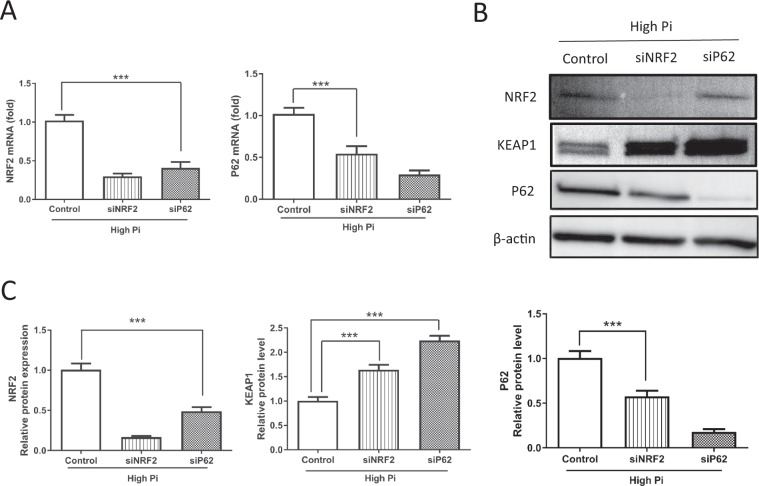
The role of KEAP1/NRF2/P62 in high Pi-induced VSMC calcification. VSMCs were transiently transfected with control, NRF2 or P62 siRNAs for 72 h and then cultured in high-Pi medium for 7 days. (**A**) The mRNA levels of NRF2 and P62 were detected by real-time PCR. (**B**, **C**) Western blot was used to evaluate the protein expression of NRF2, P62, and KEAP1. siNRF2 downregulated both the mRNA (P = 0.0008) and protein (P = 0.0005) levels of P62, while siP62 decreased the mRNA (P = 0.000029) and protein (P = 0.000037) levels of NRF2. The protein levels of KEAP1 were upregulated by siNRF2 (P = 0.0011) and siP62 (P = 0.00071) (n = 3, **P < 0.01 and ***P < 0.0001, mean ± SD).

To elucidate the interactions among NRF2, KEAP1, and P62 during VSMC calcification, the expression of these molecules was investigated after NRF2 or P62 silencing. Interestingly, NRF2 silencing markedly downregulated P62 expression at both the mRNA and protein levels, while KEAP1 protein levels were increased. In comparison, P62 silencing dramatically increased the level of KEAP1 protein, whereas it decreased NRF2 expression at the mRNA and protein levels compared with the control (Figure 7A–C), which is consistent with their known molecular interactions^23,31^. The level of KEAP1 mRNA was not affected by the siRNAs against either NRF2 or P62 (Suppl. Figure 4). These results indicate that crosstalk exists between KEAP1, NRF2, and P62 when VSMCs are exposed to high Pi concentration.

## Discussion

Vascular calcification is a highly prevalent complication in various diseases and conditions, such as chronic kidney disease, diabetes mellitus, and aging. Accumulating evidence has implicated ROS-induced oxidative stress in the progression of vascular calcification^7,32,33^. In the present study, we used an *in vitro* model of VSMC calcification induced by high Pi concentrations to reveal that high Pi concentration significantly increased intracellular ROS production, which is consistent with the finding that calcification is linked to oxidative stress^7,34^. Since the maximum physiological concentrations of phosphate and calcium are approximately 1.3 mM and 2.5 mM, respectively, cultured human VSMCs were treated with 3.0 mM phosphate and 2.7 mM calcium for 7 days to mimic hyperphosphatemia^33^, which experimentally triggers ROS production. Increased ROS production that exceeds the capacity of cellular antioxidant mechanisms is considered to be a fundamental cause of the osteogenic differentiation of VSMCs, leading to vascular calcification^7,32^. We do not know the precise mechanism involved in the induction of oxidative stress by high Pi concentrations; however, there is evidence of the effect of elevated Pi on mitochondrial function. In a previous study, it was demonstrated that high extracellular phosphate concentrations induced hyperpolarization of the mitochondrial membrane, which resulted in elevated ROS production^35^.

In our model, calcification was associated with the upregulation of osteogenic factors, such as BMP2, RUNX2/CBFA1, and osteopontin, and the downregulation of the smooth muscle cell marker α-SMA, as was previously reported^9,36^. In recent years, because of a better understanding of the underlying molecular mechanisms, therapeutic interventions that target oxidative stress represent effective strategies for treating various diseases caused by prolonged oxidative stress^37–40^. NRF2, which is suppressed by the negative regulator KEAP1, protects against oxidative stress by inducing the expression of antioxidant proteins and phase 2 detoxification enzymes in response to changes in the intracellular redox balance. According to the results of previous studies, an imbalance in the oxidant-antioxidant system caused by increased ROS production may result in cell death. NRF2 decreases intracellular ROS levels through its antioxidant activity^18,41^. In the present study, VSMC calcification induced by high Pi levels resulted in the upregulation of the transcription of NRF2 and its nuclear translocation. Additionally, the mRNA level of NRF2 target genes, including NQO-1 and HO-1, were also upregulated. These findings indicated that high Pi concentrations may stimulate antioxidant activity by increasing NRF2 expression and promoting its nuclear translocation. However, mild oxidative stress can activate a protective mechanism via the NRF2 signaling pathway, while high ROS accumulation activates a stress signal related to cell death rather than survival^42^. Additionally, according to the previous report^43^, NRF2 signaling is activated in response to ROS and regulates bone metabolism positively or negatively depending on the degree of oxidative stress. NRF2 signaling positively controls bone homeostasis by maintaining an intracellular redox balance, whereas NRF2 may negatively regulate cellular differentiation to osteoblasts through inhibition of the Runx2-dependent transcriptional activity. Thus, in this study, we hypothesize that NRF2 activation initially occurs as a response to ROS to protect from VSMC calcification, but eventually it favors calcification when ROS levels exceed the antioxidant capacity. The addition of tBHQ promotes the nuclear translocation of NRF2 and further increases the expression of its target genes, which has a protective effect against calcification. Therefore, it is possible that excessive activation of NRF2 induced by tBHQ could protect against vascular calcification.

KEAP1 has been identified as a critical repressor of NRF2 activity. Importantly, P62 not only plays important roles in removing ubiquitinated proteins but also regulates the KEAP1/NRF2 signaling pathway^26,44–46^. Crosstalk between KEAP1, NRF2, and P62 has been investigated previously^22,29,31^. P62 directly interacts with KEAP1 and disrupts the interaction between KEAP1 and NRF2, resulting in increased NRF2 activity. The accumulation of NRF2 induces the transcription of numerous cytoprotective genes, whereas the P62 gene is an NRF2 target. Consequently, NRF2 induces P62 transcription, which in turn increases NRF2 activity by inactivating KEAP1 to produce a positive feedback loop^25,47^. In the present study, we found that the KEAP1 level was markedly decreased by high Pi exposure, which is in line with the results of a previous report^48^. Interestingly, the P62 mRNA level was increased whereas the protein level was decreased. According to a previous report, P62 is an autophagic substrate that interacts with LC3 to identify polyubiquitinated protein aggregates and transport them to the autophagosome for degradation^49^. Based on a report by Tao *et al*., oxidative stress and excessive ROS accumulation can promote autophagy and induce the osteoblastic differentiation of adipose-derived mesenchymal stem cells^50^. In addition, the activation of NRF2 may induce the autophagy of VSMCs to reduce hyperphosphatemia-induced vascular calcification^51^. Therefore, it is proposed that NRF2 upregulates P62 at the mRNA level only and that along with the reduction in KEAP1 levels and the induction of high levels of Pi-induced oxidative stress, it may stimulate autophagy, thereby reducing the protein level of P62, which is consistent with the increased expression of LC3 (Suppl. Fig. 5A–C).

To gain deeper insight into the KEAP1/NRF2/P62 pathway and VSMC calcification, we used tBHQ to stimulate the KEAP1/NRF2/P62 signaling pathway in this study. tBHQ is a widely used food preservative that prevents the rancidity of lipids and is intended to be used at a level of up to 200 mg/kg of fat or oil^52,53^. In addition, tBHQ has been shown to be a well-characterized NRF2 activator in a variety of experimental settings. tBHQ activates Nrf2 through modification of the thiol groups on cysteines in Keap1, which induces antioxidant responses^30,54,55^. After treatment with different concentrations of tBHQ, ROS levels were significantly reduced in VSMCs, and there was a dose-dependent decrease in calcium deposition. In addition, compared with cells exposed to high Pi concentrations, cells exposed to tBHQ (20 μM) had significantly upregulated expression of NRF2 and P62, increased NRF2 nuclear translocation, and increased expression of the target genes NQO-1 and HO-1. These findings show that oxidative stress specifically induced by high Pi concentrations was maximally decreased by induction of the KEAP1/NRF2/P62 antioxidant system by tBHQ, which resulted in basic redox regulation. In addition, these results strongly support the hypothesis that tBHQ-induced activation of KEAP1/NRF2 signaling significantly attenuates VSMC calcification by decreasing ROS levels, which may raise the possibility of the therapeutic use. Previous studies in humans have shown that the serum concentration of tBHQ is between approximately 180 μM to 220 μM 3 h after consuming 125 mg, and the acceptable daily intake (ADI) of tBHQ is 0.7 mg/kg of body weight per day^52,56,57^. Therefore, it would be possible to reduce vascular calcification levels by administering an amount of tBHQ equivalent to the ADI to humans.

We performed siRNA-mediated silencing of NRF2 and P62 to further investigate the relationship between the KEAP1/NRF2/P62 pathway and VSMC calcification. After NRF2 and P62 knockdown, the ROS levels were increased. Indeed, knockdown of NRF2 or P62 with siRNAs has previously been shown to increase ROS levels^58,59^, which confirms our results. Meanwhile, during high Pi-induced oxidative stress, NRF2 silencing significantly decreased P62 expression at both the mRNA and protein levels, and P62 silencing reduced the expression of NRF2, which confirms that P62 is a target of NRF2 and that there is a feedback loop between them^25^. The level of KEAP1 protein was increased, while the mRNA level was not affected by NRF2 silencing. According to a previous report, decreased expression of KEAP1 attenuated the ubiquitination of NRF2 and thus increased the abundance of NRF2 in the nucleus, while KEAP1 overexpression had the opposite effect^60^. It is therefore suggested that negative regulation exist between KEAP1 and NRF2.

Furthermore, we showed that P62 silencing increased the KEAP1 protein expression level, suggesting that there may exist an interaction between P62 and KEAP1 during VSMCs calcification. This result is in agreement with some results of a previous report^23^, which showed that RNAi depletion of P62 resulted in an increase in the KEAP1 protein level by slowing its rate of degradation and concomitantly decreasing the NRF2 protein level. These findings indicate that interactions occur among KEAP1, NRF2, and P62 during the process of VSMC calcification. In addition, calcium deposition was significantly increased and tBHQ treatment lost a protective effect on calcification in the NRF2- or P62-silenced groups compared with the control groups. Consistent with our study, it has been shown that upregulation of NRF2 by dimethyl fumarate (DMF) could improve vascular calcification, while NRF2 knockdown by siRNA resulted in the increased expression of Runx2 and osteocalcin and suppressed the inhibitory effect of DMF on calcification in Rat VSMCs^61^. Overall, we concluded that the NRF2 antioxidant system is activated as a response to high Pi-induced oxidative stress and plays a protective role in calcification when activated exogenously by tBHQ.

In conclusion, our study identified a novel function of KEAP1/NRF2/P62 signaling in ameliorating high Pi-induced oxidative stress and subsequent VSMC calcification. The activation of the KEAP1/NRF2/P62 pathway by tBHQ significantly reduced high Pi-induced oxidative stress and vascular calcification, whereas NRF2 or P62 knockdown with siRNA exacerbated vascular calcification. Our findings also provide insights into the use of this system as a potential therapeutic target for vascular calcification in patients with CKD and atherosclerosis. Due to the complexity of the interactions between the KEAP1/NRF2/P62 pathway, oxidative stress, and vascular calcification, further studies are needed to provide additional information about the underlying mechanisms.

## Methods

### Reagents and antibodies

Primary antibodies against P62 (pm045), and LC3 (pm036) were purchased from MBL (Japan). An antibody against KEAP1 (#7705) was obtained from Cell Signaling Technology (Beverly, MA). An antibody against NRF2 (sc-722) was purchased from Santa Cruz. An anti-αSMA antibody (ab7817) was obtained from Abcam. Alexa Fluor 488-conjugated goat anti-rabbit, Alexa Fluor 555-conjugated donkey anti-mouse, HRP-conjugated goat anti rabbit, and HRP–conjugated rabbit anti-mouse antibodies, all primers for gene expression analyses, and Lipofectamine RNAiMAX reagents were purchased from Invitrogen. tBHQ (tertiary butylhydroquinone), anti-β-actin, DMEM (high glucose), and 2× Laemmli sample buffer were purchased from Sigma.

### Cell culture and treatment conditions

Human aortic smooth muscle cells (VSMCs) were obtained from KURABO (ks-4009, Japan). Cells were maintained in vascular cell growth medium (ks-2170s) supplemented with VSMC growth kit components comprising hEGF, hFGF-B, antibiotics (penicillin-streptomycin), and fetal bovine serum (FBS). Cells were used at passages three to five.

### Induction of calcification

VSMCs were seeded in culture dishes at a density of 1.5 × 10^5^ cells/cm^2^ and maintained in 10% FBS-DMEM (high glucose). After they become 70–80% confluent, NaH_2_PO_4_/Na_2_HPO_4_ and CaCl_2_ were added to the culture medium to final concentration of 3.0 mM and 2.7 mM, respectively, for 7 days to induce calcification. The medium was replaced every 48 h.

### Quantification of calcium deposition and Alizarin red S stainin**g**

The calcification of VSMCs was detected using Alizarin red S staining, as described elsewhere^62,63^. For the quantification of calcium deposition, cultured VSMCs were washed with PBS twice and fixed with 70% ethanol for 1 h at 4 °C. Then, VSMCs were stained with 0.2% Alizarin red S in 2% ethanol for 20 min. After four washes with distilled water and drying at 37 °C, Alizarin red S was extracted from the samples with 0.5 N HCl and 5% SDS and the absorbance was measured at 415 nm^33^.

### siRNAs transfections

Predesigned siRNAs against NRF2, P62, and a control sequence were acquired from Invitrogen. VSMCs were seeded in 6 cm culture dishes or 6-well plates 1 day prior to transfection. Cells were transfected with siRNAs targeting NRF2, P62, and the control sequence (10 nM) using the Lipofectamine RNAiMAX reagent (Invitrogen) according to the manufacturer’s protocol. Transfect efficiency was confirmed by Western blotting and real-time PCR.

### Quantitative real-time polymerase chain reaction (qRT-PCR)

Total RNA was isolated from VSMCs using a RNeasy mini kit (QIAGEN, Valencia, CA) according to the manufacturer’s protocol. Single-stranded cDNAs were synthesized with the Primescript RT master mix (Takara Bio, Shiga, Japan). The level of each mRNA was normalized to the level of the GAPDH mRNA in each sample. The comparative CT method (2^−△△CT^) was used to calculate the average fold differences between pairs of samples. Each reaction was performed in triplicate. The primers used for qRT-PCR are shown in Table 1.

**Table 1:** Primers used for Real-time PCR.

Gene	Sequence	Accession Number
**NRF2 F:** **NRF2 R:**	CACATCCAGTCAGAAACCAGTGG GGAATGTCTGCGCCAAAAGCTG	NM_001313904.1
**P62 F:** **P62 R:**	GCCAGAGGAACAGATGGAGT TCCGATTCTGGCATCTGTAG	M88108.1
**KEAP1 F:** **KEAP1 R:**	TGGCCAAGCAAGAGGAGTTC GGCTGATGAGGGTCACCAGTT	NM_203500.1
**NQO-1 F:** **NQO-1 R:**	CCTGCCATTCTGAAAGGCTGGT GTGGTGATGGAAAGCACTGCCT	NM_000903.2
**HO-1 F:** **HO-1 R:**	CCAGGCAGAGAATGCTGAGTTC AAGACTGGGGCTCTCCTTGTTGC	NM_002133.2
**BMP2 F:** **BMP2 R:**	TGACGAGGTCCTGAGCG CCTGAGTGCCTGCGATA	NM_001200.3
**osteopontin F:** **osteopontin R:**	GTTTCGCAGACCTGACATCC CATTCAACTCCTCGCTTTCC	J04765.1
**CBFA1 F:** **CBFA1 R:**	AACCCAGAAGGCAGACAGA GGCGGGACACCTACTCTCATAC	NM_00101505.1.3
**α-SMA F:** **α-SMA R:**	CAAACAGCCTCTTCAGCACA ACAGGAAGTTGGGGCTGTC	NM_001613.3
**GAPDH F:** **GAPDH R:**	GAGTCAACGGATTTGGTCGT GACAAGCTTCCCGTTCTCAG	AJ005371.1

F: forward primer, R: reverse primer.

### Extraction of nuclear and cytoplasmic proteins

VSMCs were harvested by trypsin digestion, washed twice with PBS, and pelleted by centrifugation. Nuclear and cytoplasmic extracts were isolated from the cells using the NE-PER Nuclear and Cytoplasmic Extraction Reagent Kit (Thermo Scientific), according to the manufacturer’s instructions. The nuclear protein level was measured as the relative ratio to histoneH1, a marker of the nuclear fraction.

### Western blot

Cultured VSMCs were quickly rinsed twice with ice-cold PBS and solubilized with 4% sodium dodecyl sulfate. Lysates were placed on ice, sonicated for 5 s, and boiled at 95 °C for 5 min. Proteins were separated using SDS-polyacrylamide gel electrophoresis, transferred to polyvinylidene difluoride membranes, blocked with 5% skim milk in TBS, and then incubated with primary antibodies overnight at 4 °C. After an incubation with secondary antibodies, proteins were detected using ECL (GE Healthcare). β-Actin was used as a control to normalize total protein levels. Anti-NRF2, anti-P62, anti-KEAP1, anti-LC3, and anti-β-actin antibodies were used as primary antibodies.

### Immunofluorescence staining

VSMCs were grown on glass chamber slides (LAB-TEK), treated with or without calcification medium, and fixed with cold acetone for 15 min. After two washes with PBST, cells were permeated with 0.1% Triton X-100 in PBS containing 1% BSA and blocked with 5% BSA in PBS for 1 h. Cells were then incubated with primary antibodies against NRF2, KEAP1, P62, and LC3, overnight at 4 °C. After washes, cells were incubated with an Alexa Fluor 488-conjugated goat anti-rabbit secondary antibody for 1 h at RT. Cells were then incubated with an antibody against αSMC for 1 h and finally incubated with an Alexa Fluor 555-conjugated donkey anti-mouse secondary antibody for 1 h. Nuclear staining was performed using VECTASHIELD Hard Set mounting medium containing 4,6-diamidino-2-phenylindole (DAPI) (Vector Laboratories, Burlingame, CA). Cells were observed under a fluorescence microscope.

### ROS assay

Cellular ROS levels were measured using the cell-permeable reagent 2′,7′-dichlorofluorescein diacetate (DCFDA, also known as H2DCFDA, ab113851, Abcam, USA). Briefly, cells were seeded in triplicate wells of in 96-well plates at a density of 0.25 × 10^5^ cells/well in 100 μL of media and allowed to grow overnight. Following transfection and/or treatments, cells were washed with 1 × buffer once and maintained in 100 μL of 1 × buffer. A 20 mM stock solution of DCFDA was added to each well and incubated for 45 min at 37 °C. Fluorescence signal intensities indicating ROS levels were recorded by measuring the absorbance using a 96-well fluorescent multiplate reader (SH-9000, Corona, Japan) using excitation and emission wavelengths of 485 nm/535 nm. Cells in the same wells were stained with Coomassie brilliant blue (Sigma) for 1 h to normalize the fluorescence signal. After washes with distilled water, a 10% sodium dodecyl sulfate (SDS) solution was added to release the absorbed dye and incubated for 10 min while shaking. The absorbance values were recorded at 595 nm using a multiplate absorbance reader (SH-9000, Corona, Japan). The data were analyzed after normalizing the fluorescence values.

### Statistical analysis

The data are presented as means ± SD. All experiments were performed a minimum of three times using independent populations of cells and ran. The significance of the differences between 2 groups was analyzed using Student’s t test and values from more than 3 groups were analyzed by a one-way analysis of variance (ANOVA) with Tukey’s post hoc test. Nonsignificant differences were noted ns. P < 0.05 was considered statistically significant.

## Supplementary information

Dataset

## Data Availability

All data generated and analized during this study are included in this published article and is supplementary information files.
