# Supplementary material for: Activation of KEAP1/NRF2/P62 signaling alleviates high phosphate-induced calcification of vascular smooth muscle cells by suppressing reactive oxygen species production
Source: Sci Rep. 2019 Jul 17;9:10366. doi: 10.1038/s41598-019-46824-2 (PMC6637199; doi:10.1038/s41598-019-46824-2)
Supplement: Supplementary file 1 — Dataset [file 41598_2019_46824_MOESM1_ESM.pdf]

Activation of KEAP1/ NRF2/P62 signaling alleviates high phosphate-induced calcification of vascular smooth muscle cells by suppressing reactive oxygen species production

Ran Wei, Mayu Enaka, and Yasuteru Muragaki

Department of Pathology, Wakayama Medical University School of Medicine

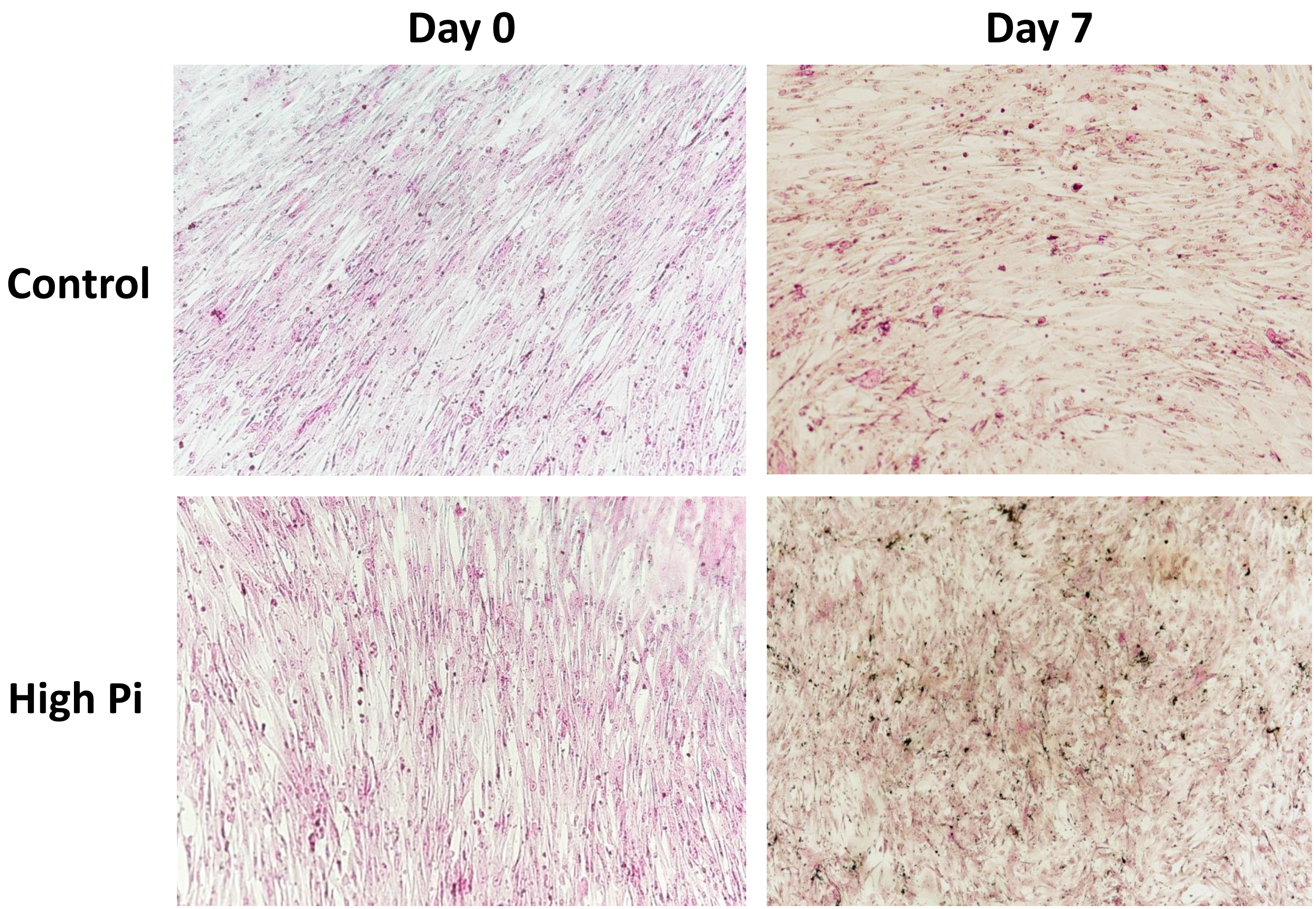

Supplementary figure 1
VSCM calcification by determined by von Kossa staining. VSMCs were cultured in either control or high Pi medium for 7 days and stained with von Kossa.

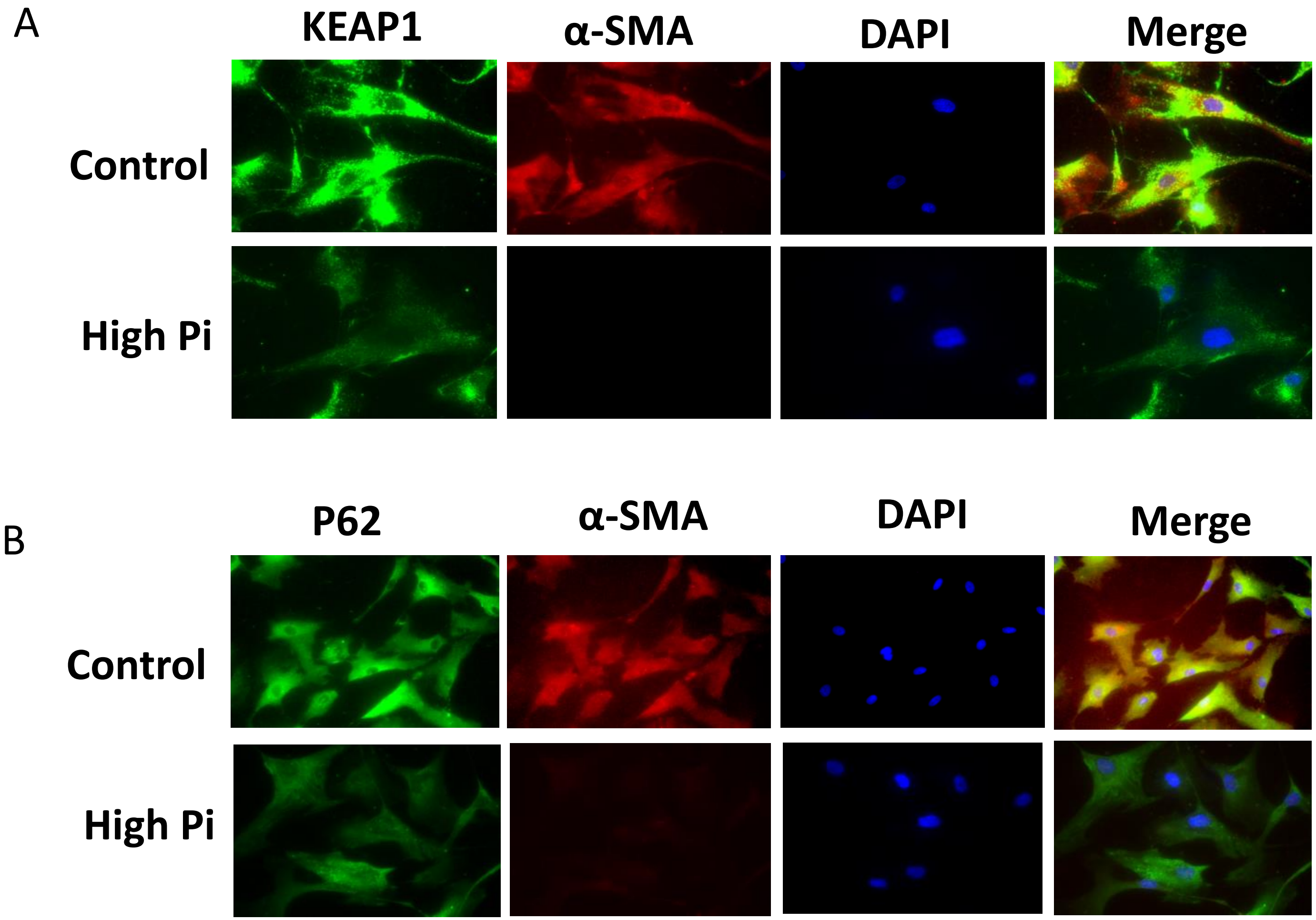

Supplementary figure 2

Expression of P62 and KEAP1 in VSMCs exposed to high Pi. (A, B) VSMCs were cultured on glass chamber slides, incubated in the presence or absence of high Pi for 7 days. The cells were fixed with cold acetone and reacted with specific primary antibodies against KEAP1, P62 and α-SMA.

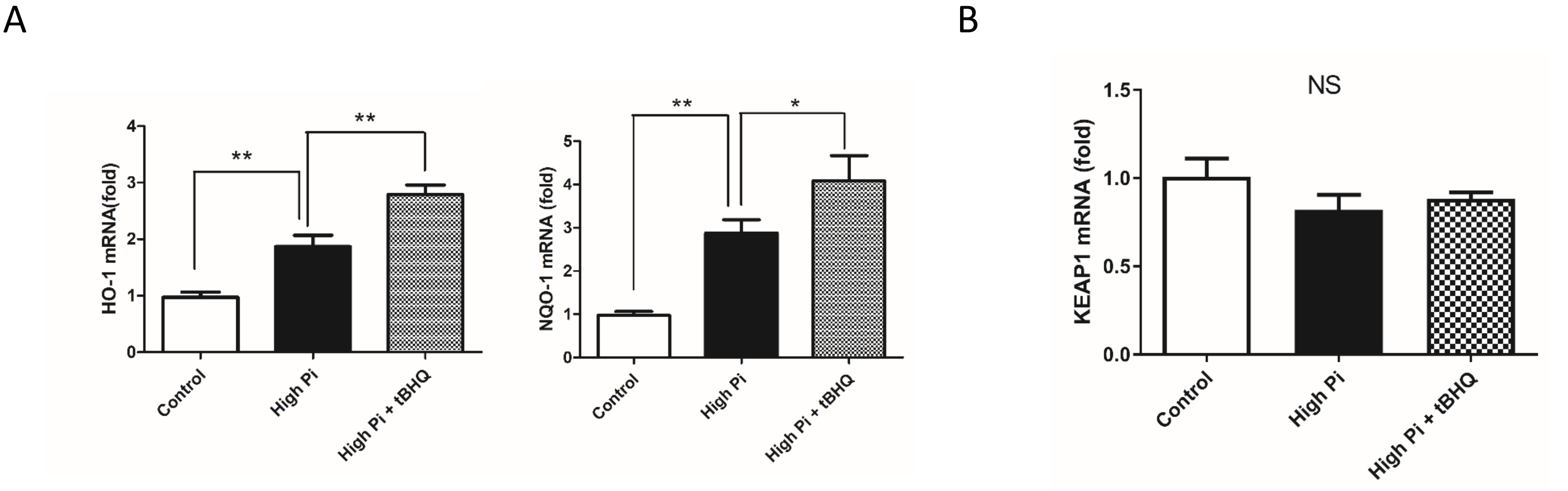

Supplementary figure 3

tBHQ ameliorates VSMCs calcification via an increase in NRF2 nuclear translocation and the expression of the downstream target genes. VSMCs were cultured in control medium, high Pi medium with or without tBHQ (20 μM) for 7 days. (A) mRNA expression of HO-1 and NQO-1 was detected by real-time PCR. (B) mRNA expression of KEAP1 was not affected by the addition of tBHQ. (n=3, data are expressed as means ± SD, *P<0.05,**P<0.01)

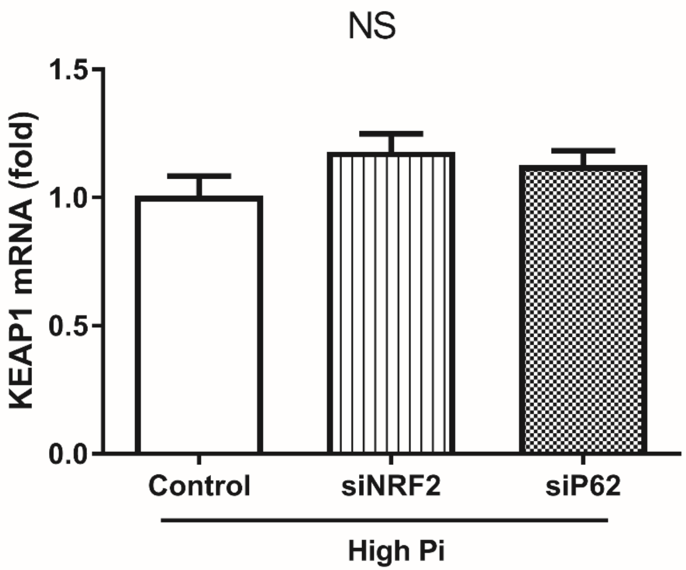

Supplementary figure 4

Crosstalk between NRF2, P62, and KEAP1 in VSMCs exposed to high Pi. KEAP1 mRNA level was analyzed by Real-time PCR.

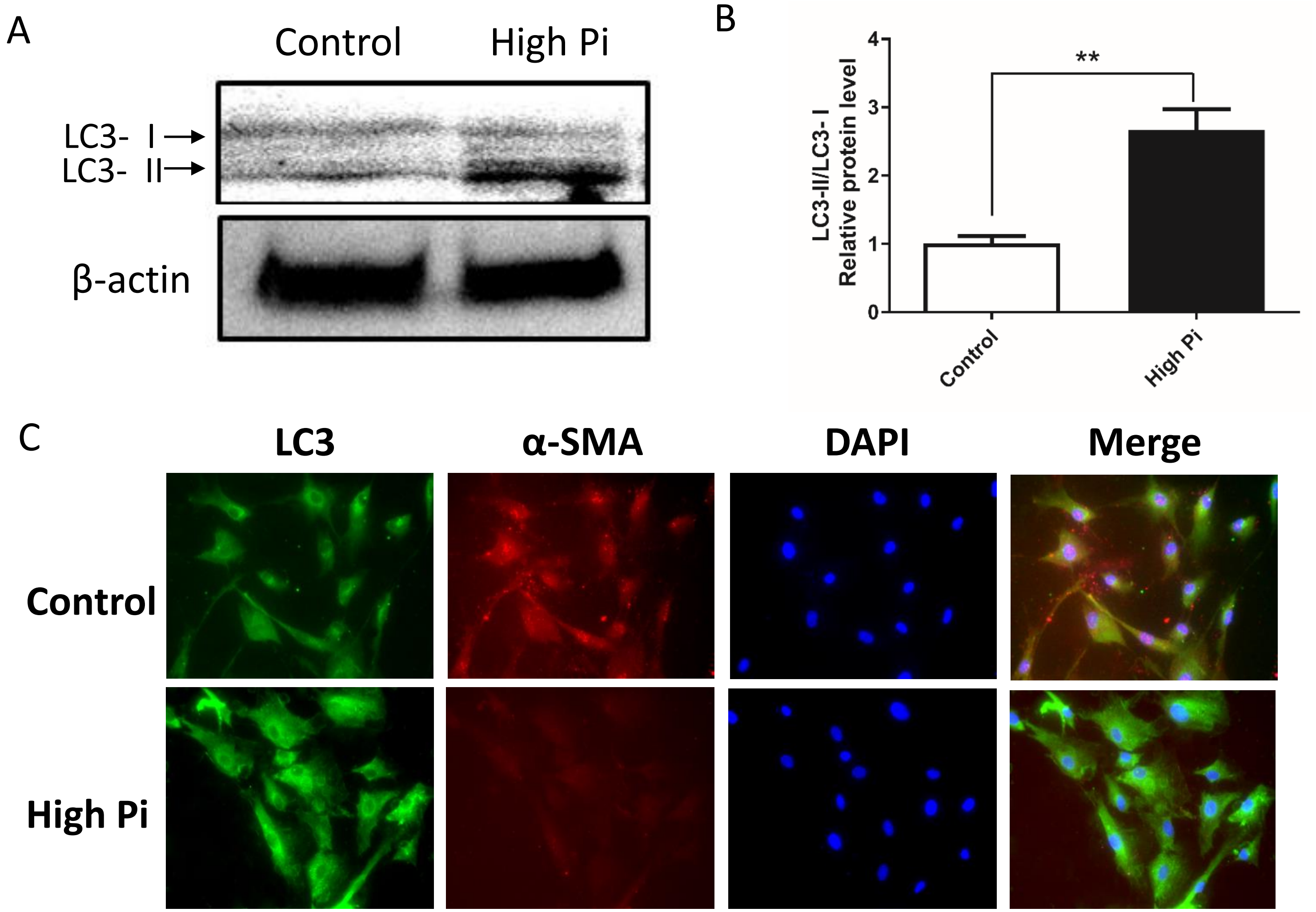

Supplementary figure 5

High Pi-induced oxidative stress stimulates autophagy. VSMCs were cultured in control medium, high Pi medium with or without tBHQ (20 μM) for 7 days. (A-B) LC3 expression was detected by Western blot and the densitometric quantification was shown. (n=3, data are expressed as means ± SD, **P<0.01). (B) Localization of LC3 by immunofluorescence staining.

Original whole panel for each western blot

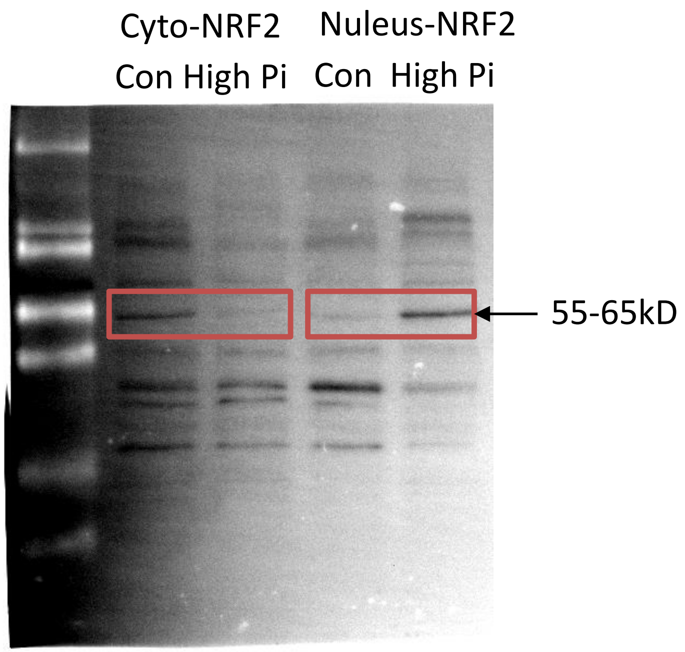

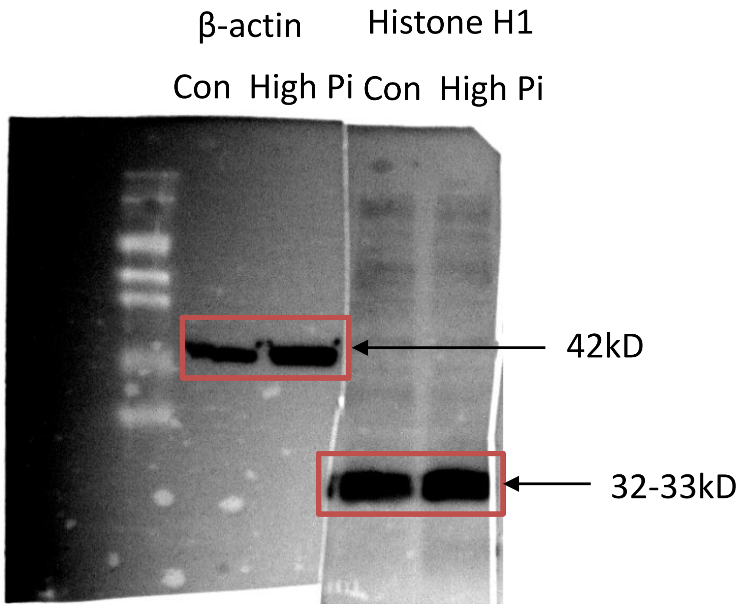

Fig 2B

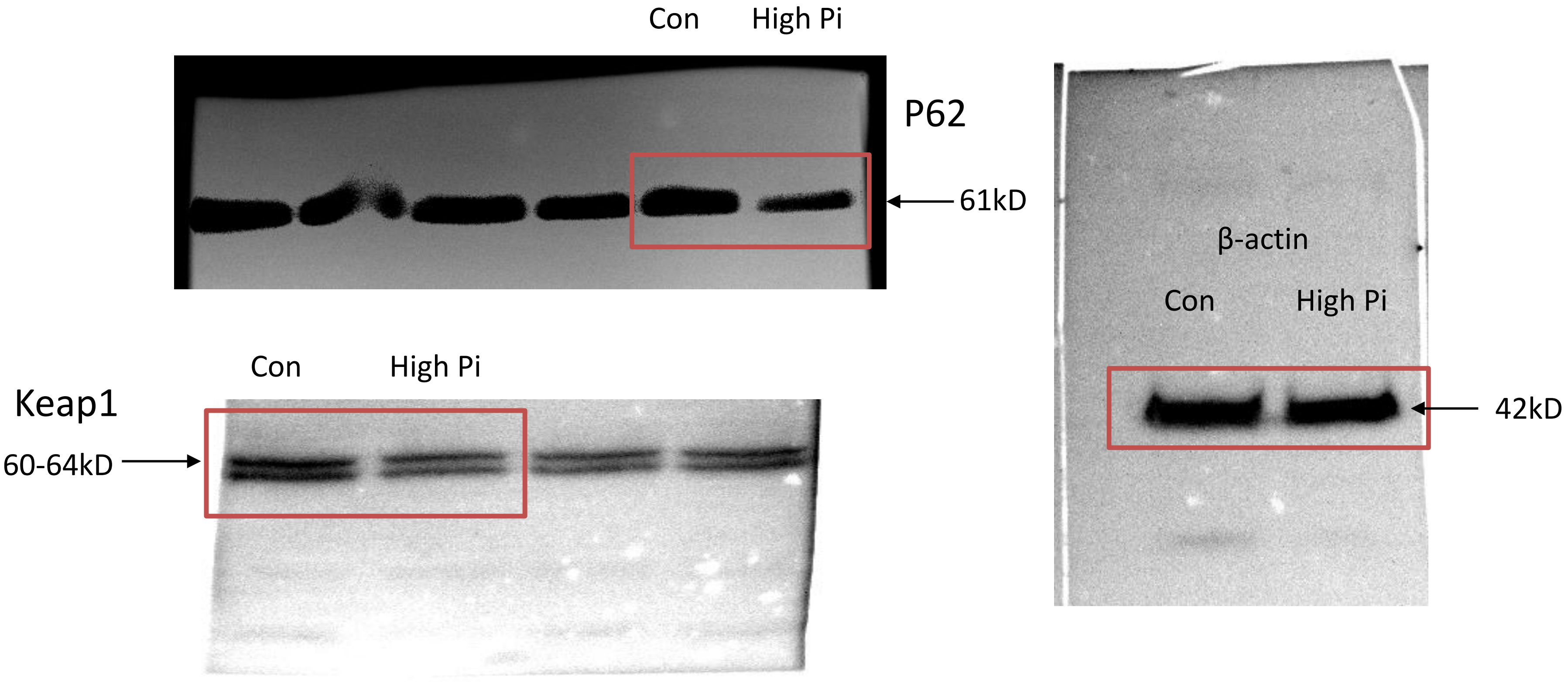
Con
High Pi
P62
61kD
Keap1
Con
High Pi
60-64kD
β-actin
Con
High Pi
42kD

Fig 3B

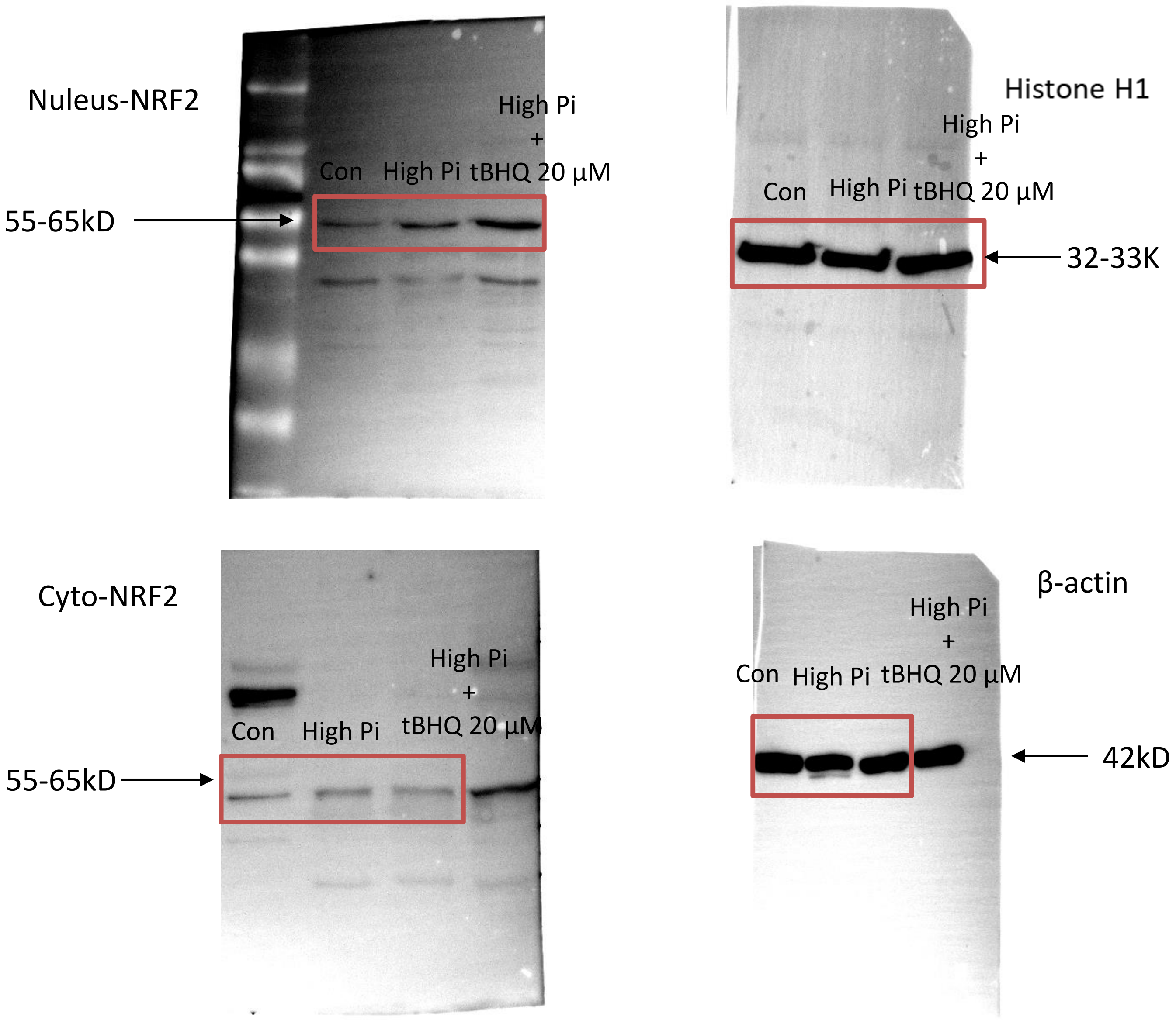
Nuleus-NRF2
Con
High Pi
High Pi + tBHQ 20 µM
55-65kD
Histone H1
Con
High Pi
High Pi + tBHQ 20 µM
32-33K
Cyto-NRF2
Con
High Pi
High Pi + tBHQ 20 µM
55-65kD
β-actin
Con
High Pi
High Pi + tBHQ 20 µM
42kD

Fig 5B

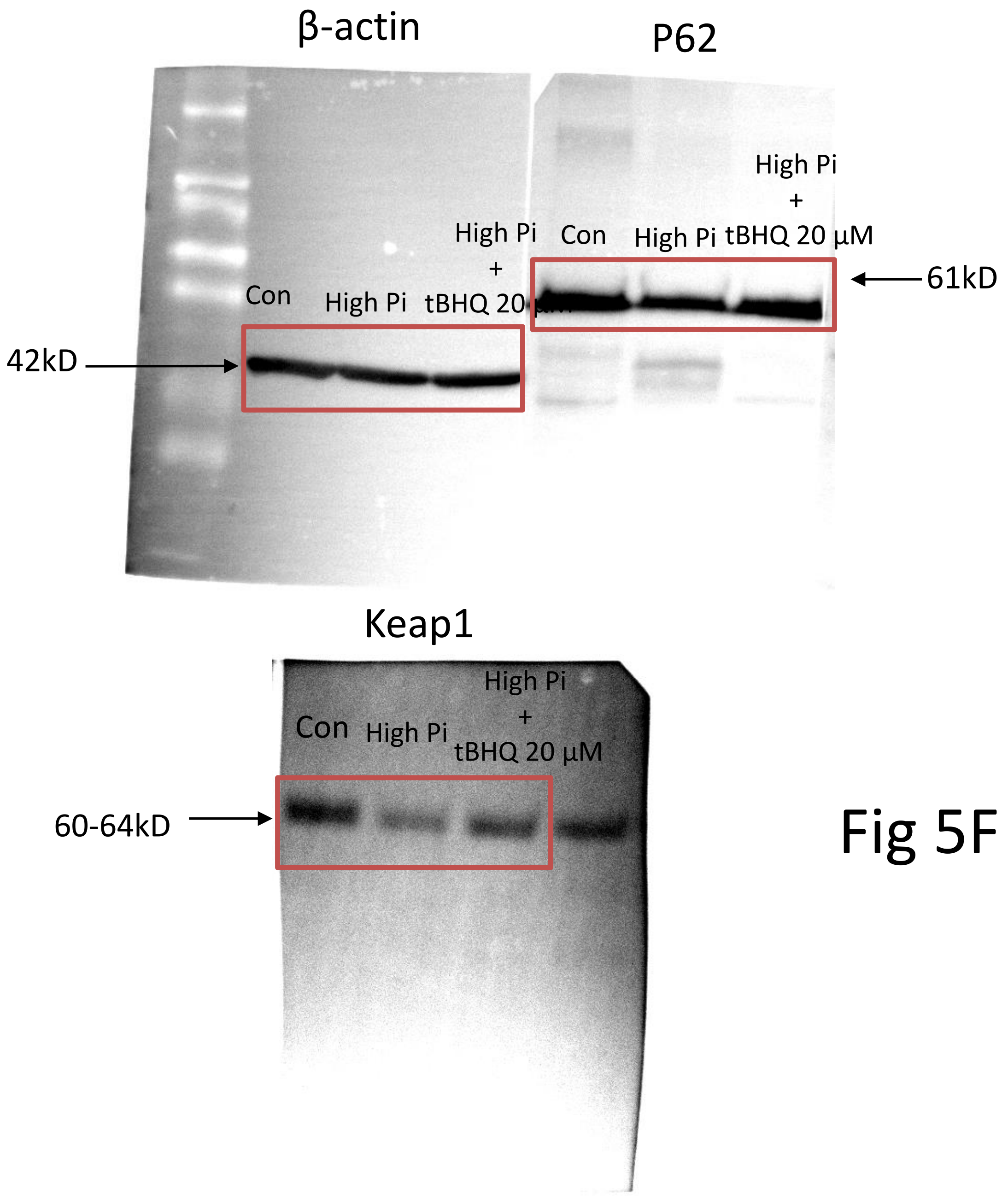
β-actin
P62
Con
High Pi
High Pi
+
tBHQ 20 μM
42kD
Con
High Pi
High Pi
+
tBHQ 20 μM
61kD
Keap1
Con
High Pi
High Pi
+
tBHQ 20 μM
60-64kD

Fig 5F

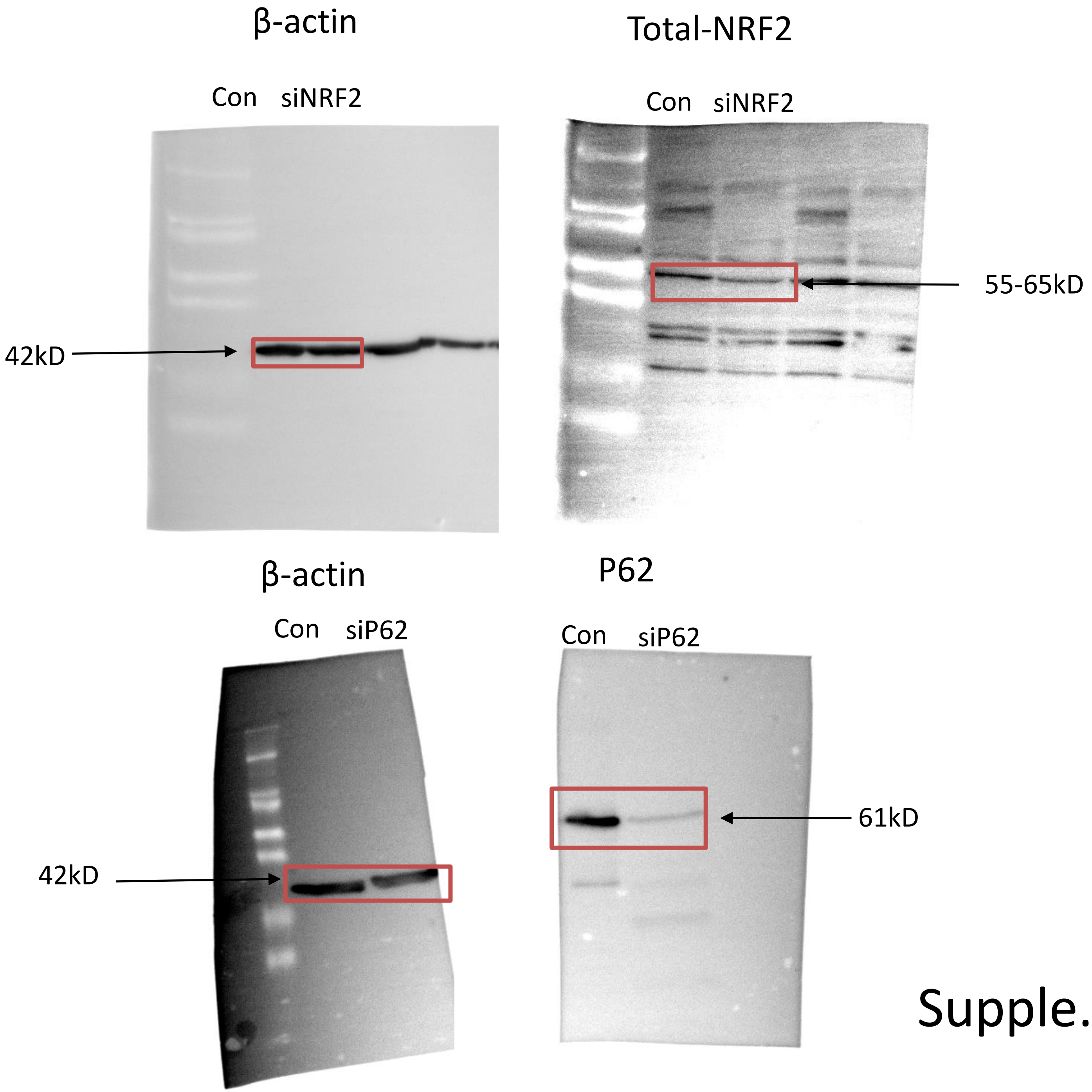
β-actin
Con siNRF2
42kD
Total-NRF2
Con siNRF2
55-65kD
β-actin
Con siP62
42kD
P62
Con siP62
61kD

Supple.Fig4

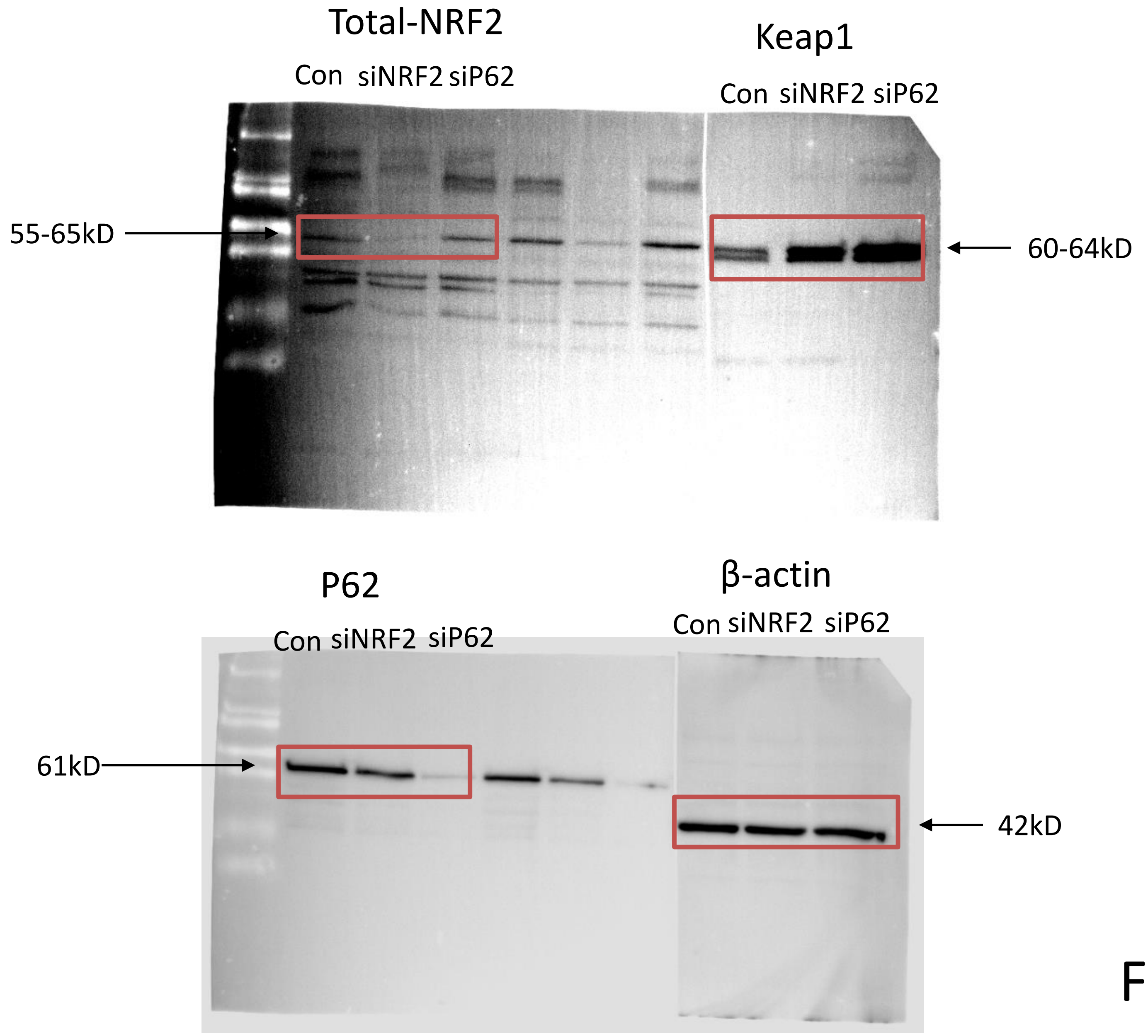
Total-NRF2
Con siNRF2 siP62
Keap1
Con siNRF2 siP62
55-65kD
60-64kD
P62
Con siNRF2 siP62
β-actin
Con siNRF2 siP62
61kD
42kD

Fig6B

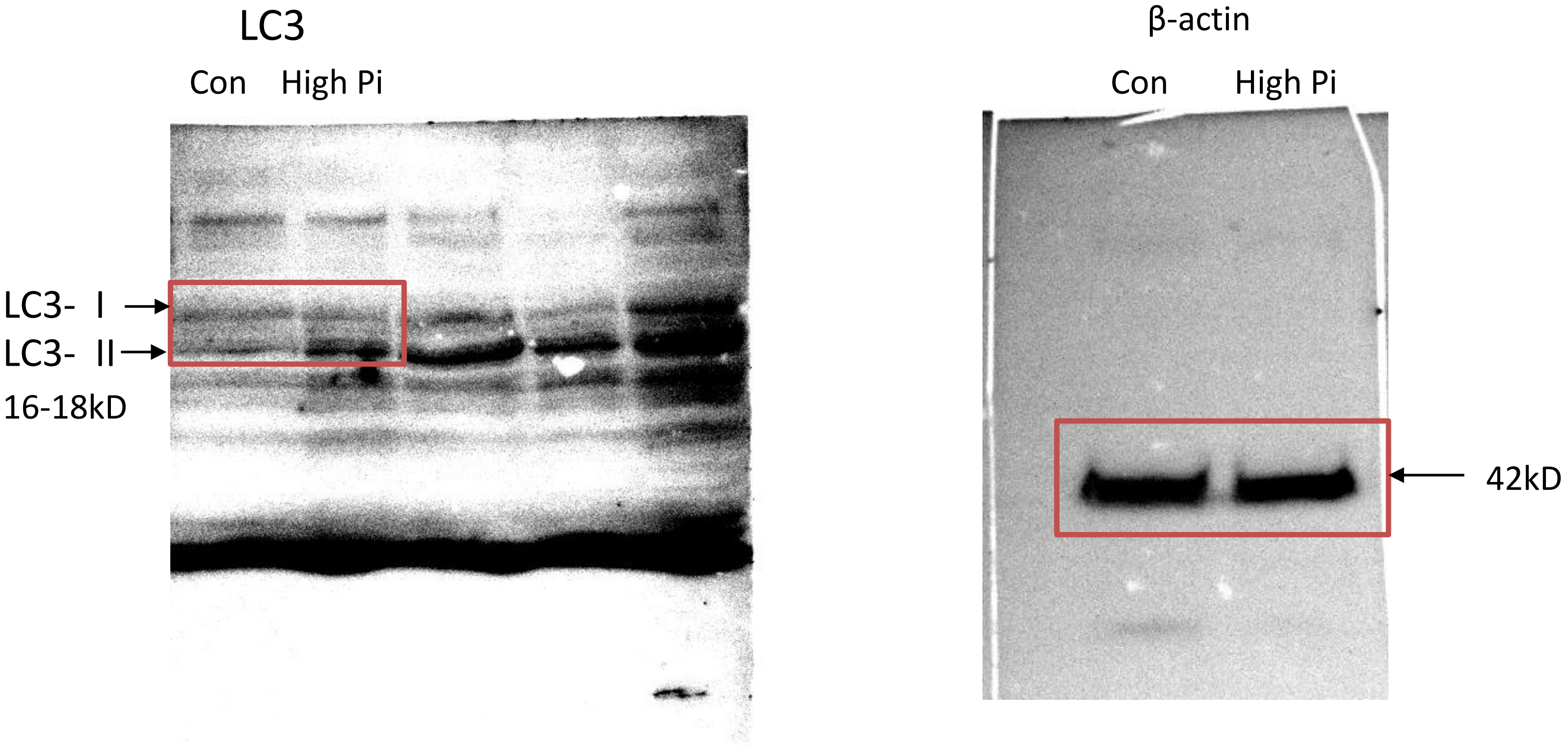

Supple.Fig6
